# Iron Deficiency in Patients with Advanced Heart Failure

**DOI:** 10.3390/medicina58111569

**Published:** 2022-10-31

**Authors:** Maria Bakosova, Jan Krejci, Julius Godava, Eva Ozabalova, Hana Poloczkova, Tomas Honek, Peter Hude, Jan Machal, Helena Bedanova, Petr Nemec, Lenka Spinarova

**Affiliations:** 1 1st Department of Internal Medicine—Cardioangiology, St. Anne’s University Hospital, 60200 Brno, Czech Republic; 2 Faculty of Medicine, Masaryk University, 60200 Brno, Czech Republic; 3 International Clinical Research Center, St Anne’s University Hospital, 60200 Brno, Czech Republic; 4 Center for Cardiovascular and Transplant Surgery, 65691 Brno, Czech Republic

**Keywords:** iron deficiency, anaemia, advanced heart failure, ferritin, transferrin saturation

## Abstract

*Background and Objectives*: Iron deficiency (ID) is a common comorbidity in patients with heart failure. It is associated with reduced physical performance, frequent hospitalisations for heart failure decompensation, and high cardiovascular and overall mortality. The aim was to determine the prevalence of ID in patients with advanced heart failure on the waiting list for heart transplantation. *Methods and Materials*: We included 52 patients placed on the waiting list for heart transplantation in 2021 at our centre. The cohort included seven patients with LVAD (left ventricle assist device) as a bridge to transplantation implanted before the time of results collection. In addition to standard tests, the parameters of iron metabolism were monitored. ID was defined as a ferritin value <100 µg/L, or 100–299 µg/L if transferrin saturation (T-sat) is <20%. *Results*: ID was present in 79% of all subjects, but only in 35% of these patients anaemia was expressed. In the group without LVAD, ID was present in 82%, a median (lower–upper quartile) of ferritin level was 95.4 (62.2–152.1) µg/mL and mean T-sat was 0.18 ± 0.09. In LVAD group, ID was present in 57%, ferritin level was 268 (106–368) µg/mL and mean T-sat was 0.14 ± 0.04. Haemoglobin concentration was the same in patients with or without ID (133 ± 16) vs. (133 ± 23). ID was not associated with anaemia defined with regard to patient’s gender. In 40.5% of cases, iron deficiency was accompanied by chronic renal insufficiency, compared to 12.5% of the patients without ID. In the patients with LVAD, ID was present in four out of seven patients, but the group was too small for reliable statistical testing due to low statistical power. *Conclusions*: ID was present in the majority of patients with advanced heart failure and was not always accompanied by anaemia and renal insufficiency. Research on optimal markers for the diagnosis of iron deficiency, especially for specific groups of patients with heart failure, is still ongoing.

## 1. Introduction

Iron deficiency (ID) and anaemia are common comorbidities in patients with heart failure, which can occur in up to the half of patients with heart failure. These conditions are associated with decreased performance status, frequent hospitalizations for heart failure decompensation, and high mortality from cardiovascular causes [1,2]. ID does not always manifest as anaemia and can often be present in individuals with normal haemoglobin levels. According to international data, nearly 1.2 billion people suffer from ID anaemia. Nearly twice as many individuals may have ID without having anaemia [3,4].

ID is associated with reduced bioenergetic reserve in organs requiring a high energy supply, such as a heart muscle [5]. Iron is involved in a number of energy metabolism processes in addition to haemoglobin formation. The body contains approximately 3–4 g of iron, which is overwhelmingly recycled and only a small proportion is replenished by absorption from the gastrointestinal tract. Iron is transported in the blood by binding to the protein transferrin, whose binding capacity is usually saturated from 20–40%. In the cells, iron is stored by binding to ferritin. Iron metabolism in body is regulated by the hepatic hormone hepcidin. It is a hormone secreted by liver in response to increased iron or inflammation and it reduces iron absorption and internal recycling via the transmembrane protein ferroportin [6,7,8].

Two forms of ID are recognized: absolute (true) ID and functional ID. Absolute ID is defined by severely reduced or absent iron stores in bone marrow, liver, and spleen. Functional ID is defined by normal or increased total body iron stores which are unavailable for incorporation into erythroid precursors for erythropoiesis [9]. Functional ID is mainly due to increased levels of hepcidin which reduce the ability to recruit iron stores from reticuloendothelial cells and hepatocytes for erythropoiesis [10].

ID in patients with chronic heart failure is caused mostly by limited internal recycling of the iron or reduced iron intake. In heart failure, inappetence is often present and iron absorption is also decreased, further exacerbating ID. Occult blood loss may contribute to iron loss, which is potentiated by anticoagulation and especially intensive antiplatelet therapy, which is given to an increasing number of patients with cardiovascular diseases [11].

These is also special group of patients with left ventricle assist device (LVAD) implanted. In this population, a high prevalence of ID anaemia was demonstrated in previous studies. In contrast to the positive effects like improving kidney function and gut absorption, we must account for presence of haemolysis, both anticoagulation and antiplatelet therapy and minor GIT bleeding which counteract the benefits of improved perfusion given by the presence of an LVAD [12,13].

There is no doubt about the importance of this topic, but in practice we encounter problems in determining the presence of ID, because the parameters used are widely influenced by the current state of the organism. Various ferritin cut-off values have been recommended to help detect ID in different patient populations, such as in those with heart failure and chronical kidney diseases or inflammatory bowel disease [14]. Low-grade inflammation in chronic inflammatory conditions is enough to disrupt iron metabolism by increasing hepcidin but does not necessarily correlate with inflammatory markers. Moreover, other mechanisms put patients with chronic inflammation at higher risk of ID and underscore the need to make a correct diagnosis despite interference in iron parameters. There is a general consensus that the usual ferritin cut-off of 30 μg/L is inappropriate in the presence of a chronic inflammatory conditions, but the recommended ferritin values range is between 50 and 500 μg/L across guidelines [15].

Based on previous studies, in patients with heart failure, ID has been defined as the presence of either a serum ferritin concentration <100 µg/L or 100–299 µg/L if the transferrin saturation (T-sat) is <20% [16,17,18].

The 2016 ESC guidelines for the diagnosis and treatment of heart failure, based on the FAIR-HF and CONFIRM-HF studies, already stated that in patients with the abovementioned ferritin or transferrin saturation values, administration of ferric carboxymaltose (FCM) should be considered for symptom reduction, improve performance status and quality of life in class IIa recommendations [19]. The 2021 ESC guidelines for the diagnosis and treatment of heart failure clearly recommend that all patients with heart failure should be examined for the presence of ID [20]. The earlier recommendations have been expanded with findings reflecting the results of the AFFIRM-AHF study, which focused on patients after acute decompensation of heart failure with ID present.

The results of the AFFIRM-AHF study confirmed that the addition of FCM to standard treatment for heart failure provided a statistically significant benefit expressed by a 21% reduction in the risk of the primary endpoint. This result was driven mainly due to a 26% reduction in the risk of hospitalization for heart failure, whereas the difference in mortality from cardiovascular cause was not statistically significant (13.8% in the FCM-treated group vs. 14.2% in the placebo arm).

## 2. Study Objectives

The study focused on the evaluation of the presence of ID in patients with advanced heart failure. Additionally, the study aimed at differences in several laboratory markers, NYHA status, left ventricular ejection fraction and the presence of anaemia in the groups of patients with heart failure with and without ID.

## 3. Methods

### 3.1. Study Population

This retrospective study was conducted in the St Anne’s University Hospital Brno, 1st Department of Internal Medicine—Cardioangiology from January 2021 to December 2021. All consecutive 52 patients placed on the waiting list for heart transplantation were included in this evaluation. Within this group, in 7 patients was LVAD already implanted as a bridge to transplantation. The shortest time from LVAD implantation surgery to laboratory tests collection was 8 weeks.

All patients had advanced heart failure with severely limiting symptoms consistent with an indication for heart transplantation. All patients had a comprehensive medical evaluation with clinical and laboratory assessments including echocardiography, electrocardiography and right heart catheterization. In study we focused on blood count, creatinine, serum N-terminal pro-B-type natriuretic peptide (NT-proBNP), CRP, serum ID tests and anticoagulation or antiplatelet therapy.

### 3.2. Inclusion Criteria

Consecutive patients with advanced heart failure who fulfilled the indication criteria for inclusion on the waiting list for heart transplantation or LVAD implantation were included in the study.

Exclusion criteria were disorders that significantly limit the prognosis or serious extracardiac diseases (infections, malignancies, peptic ulcer disease, severe kidney, liver or lung dysfunction, generalized atherosclerosis with hemodynamically significant involvement of the arterial system, diabetes mellitus with organ complications, drug or alcohol abuse, psychosocial instability, obesity with BMI > 35 kg/m^2^, age over 65–70 years, taking into account the biological status of each patient) [19].

In the process of research, we decided to explore the small group with LVAD implanted also separately because of noticed differences of parameters of ID and differences in pathophysiology of iron deficiency and anaemia in this group.

### 3.3. Biological Tests and Assessment of ID Status and Anaemia

ID was diagnosed by serum levels of ferritin and transferrin saturation (T-sat). ID was diagnosed when patients had serum ferritin <100 μg/L, or serum ferritin between 100 and 299 μg/L with transferrin saturation <20% as defined by the 2021 ESC HF guidelines [10,11,12]. Anaemia was defined as haemoglobin levels <130 g/L for men and <120 g/L for women, as recommended by the World Health Organization [21].

### 3.4. Materials and Measurements

Presented echocardiography parameters are in accordance with Lang’s Recommendations for Cardiac Chamber Quantification by Echocardiography in Adults [22].

All laboratory parameters were evaluated in a certified FNUSA laboratory according to established methodologies. Ferritin was detected by chemiluminescence immunoassay on microparticles by the Ferritin Reagent Kit from the Abbot company. Serum iron by the direct photometric assay by the Roche corporation, transferrin was detected immunochemically by the Roche kit and T-sat was calculated from the concentration of transferrin and serum iron. Hemoglobin was measured via absorbance photometry using a Sysmex reagent. All test were taken in morning hours before meals.

### 3.5. Statistical Analysis

Continuous variables with normal distribution were expressed as mean ± standard deviation (SD), those with different distribution as median (lower–upper quartile) and dichotomous data as numbers with percentages.

Continuous data of two groups of patients were compared using two-sample Student’s *t*-test. The test was applied after checking for normal distribution by visual inspection of histograms or normal probability plots, and by Kolmogorov–Smirnov test. Some variables (ferritin NT-proBNP, creatinine) showed log-normal distribution and data transformation was performed prior to the testing. Binary data were compared using two-tailed Fisher exact test. The correlations of two continuous parameters in one group are expressed as Pearson correlation coefficient with a respective *p*-value (again, the data were log-transformed, when needed). Power of the test was considered in order to determine the effect size that could be proven as statistically significant in a given sample size. The estimations are given for 60% power and for 80% power.

All data were obtained from medical files collected in our department. The statistical analyses were performed using Statistica (version 14.0. TIBCO Software Inc., 2020, Palo Alto, CA, USA) software. A *p*-value < 0.05 was considered as statistically significant.

## 4. Results

### 4.1. Patient Characteristics

Patients’ data were collected from January 2021 to December 2021 from the medical reports of patients placed on the waiting list for an orthotopic heart transplantation (OHT). All patients signed informed consent to the use of their medical data for scientific purposes, and this study was approved by the Ethics Committee of the St. Anne’s University Hospital under the number 8G/2022.

The whole cohort of 52 patients included 7 patients with implanted LVAD as a bridge to transplantation before the time of results collection and another 45 patients without LVAD implanted (Table 1).

The median age in the patients without LVAD was 59 years (56.1 ± 10.8) and 54 (54.3 ± 5.6) for patients in those with implanted LVAD. Women represented 25% of all patients. There was no woman in LVAD group.

**Figure 1:**
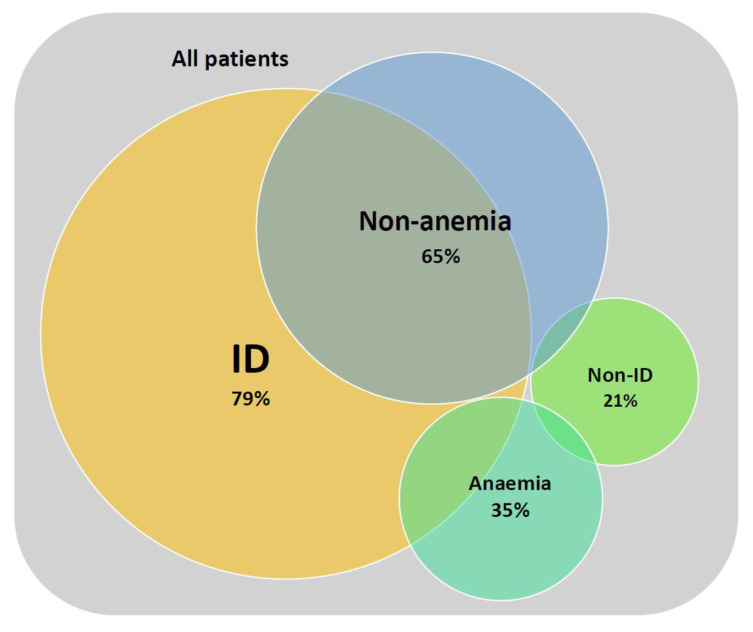
Prevalence of ID and anaemia in whole study group.

ID was present in 79% of all subjects, but only in 35% of these patients anaemia was expressed. In the group without LVAD, ID was present in 82%, in LVAD group in 57% (Figure 1).

The baseline characteristics of study groups are shown in Table 1.

### 4.2. Prevalence of ID among Heart Failure Patients without LVAD

ID was diagnosed in 37 patients on waiting list for heart transplantation which represents 82% of this group; 8 patients (18%) had no proven ID. Table 2 shows the results calculated in relation to iron deficiency.

### 4.3. Iron-Related Parameters in Patients on Waiting List

Focusing on the patients without LVAD, correlations of laboratory parameters shown in Table 1 (i.e., NT-proBNP, creatinine, haemoglobin, serum ferritin, transferrin saturation, serum iron and CRP) were performed. Some of the iron-related parameters showed correlations: there was a weak to moderate positive correlation between transferrin saturation and serum ferritin (r = 0.37, *p* = 0.01) and a very strong correlation between transferrin saturation and serum iron (r = 0.88, *p* < 0.001). Serum iron correlated positively with haemoglobin value (r = 0.38, *p*= 0.009) and negatively with NT-proBNP (r = −0.45, *p* = 0.002) and CRP (r = −0.39, *p* = 0.008). In the case of CRP and haemoglobin, serum iron was the only other marker, where the correlation was statistically significant. Creatinine correlated with serum iron (r = −0.30, *p* = 0.048) and with NT-proBNP (r = 0.38, *p* = 0.01). All the other correlations yielded results with *p*-values > 0.10 and r < 0.25.

**Figure 2:**
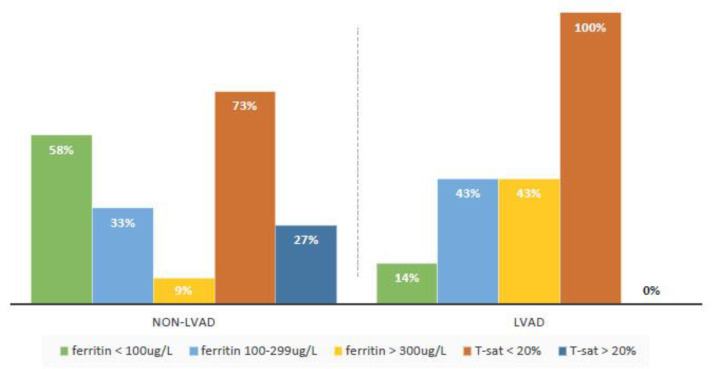
Percentage of iron deficiency parameters in non-LVAD and LVAD patients.

If we compare this numbers with a small group of patients with LVAD, some iron-related parameters differed between the qroups: serum ferritin (higher in LVAD group, *p* = 0.019) and serum iron (lower in LVAD group, *p* = 0.006), while T-sat did not differ between the groups, what we can state like a very interesting result. Because of the prevalence of iron deficiency by conventional definition was lower in patients with LVAD, it may suggest the question whether the criteria based on ferritin level are appropriate in this patient group and whether T-sat as an isolated marker would not be a more accurate marker of ID in patients with LVAD (Figure 2).

This hypothesis is supported by other findings: differences in levels of haemoglobin between both groups (lower in LVAD group, *p* < 0.001) and the prevalence of anaemia (higher in LVAD group, *p* < 0.001).

### 4.4. Power of the Test

The power analysis was employed to determine the magnitude of effect that could have been identified as statistically significant: for correlations and given the power of 80%, α = 0.05 and *n* = 45, the test was able to identify |r| > 0.40 as significant. With the power of 60%, the correlation identified as statistically significant was |r| > 0.33. In the case of comparison of haemoglobin concentration (*n*1 = 37, *n*2 = 8, α = 0.05, σ = 20 g/L), the test was able to identify a difference of 23 g/L with 80% power and 18 g/L with 60% power. Similarly in other parameters, the sample size was sufficient to reveal moderately high effects in whole sample or specifically in the patients without LVAD, but was not sufficient for reliable analyses within the LVAD group.

## 5. Discussion

We found a high prevalence of iron deficiency (ID) in population of patients with advanced heart failure, which was present in 79% in whole cohort. While collecting the results, we noticed some differences in the LVAD group, and when we separated this small group, we found some differences between the groups. ID was present in 82% in the group without LVAD implanted.In contrast, in the LVAD group ID was present only in 57%. This finding seems to be quite surprising in view of the previous knowledge of the presence of ID anaemia in patients with LVAD [10,11].

Patients in the non-LVAD group could be reliably divided according to ferritin and T-sat levels into groups; without and with ID (*p*-value for both differences for ferritin and T-sat was <0.001).

In contrast, in the LVAD group, values of ferritin and T-sat were discordant; all patients had low T-sat values, but the ferritin value was higher in whole LVAD group comparing with patients without LVAD (268 vs. 95 μg/L in non-LVAD group, *p*-value 0.019). In 3 patients, the ferritin level exceeded 300 μg/L, which excludes these patients from the ID category according to the current definition, even though they would fall into the ID category according to the value of T-sat.

Next, serum iron levels follow the differences in ferritin and T-sat levels: serum iron is lower in the entire LVAD-implanted group (11 µmol/L in non-LVAD group vs. 7.8 µmol/L in LVAD group, *p*-value 0.006). Correlation between serum iron and T-sat was found in previous studies and T-sat <20% with serum iron <13 µmol/L was associated with higher 5-year mortality [23].

Our results support opening a debate on the reliability of ferritin as a basic parameter for the determination of ID; it may raise the hypothesis, that the cause of this finding is not the real absence of ID in patients with LVAD, but the result of ID presence is false negative according to the diagnostic parameters used so far, especially due to ferritin level assessment. Although ferritin is the most specific marker, it is less sensitive because its blood levels also increase with infection or any type of chronic inflammation.

Our findings can support recent study, that used the gold standard of bone marrow staining to quantify an amount of iron and claims, that T-sat ≤19.8% or a serum iron ≤13 µmol/L shows the best performance in selecting patients with ID and validated the currently used T-sat cut-off of <20% for the identification of ID in HF patients and call into question the value of serum ferritin in the assessment of ID [24]. Additionally, this study suggests, that a ferritin in the range of 50–500 μg/L corresponds to normal bone marrow iron content, and T-sat helps determine which patients will benefit more from iron supplementation. A normal T-sat of 20 to 45% is associated with adequate iron stores in most chronic inflammation conditions. T-sat tend to be reduced in inflammation, probably due to elevated hepcidin levels [6,15].

Another study supporting the hypothesis that T-sat is a more appropriate marker for ID is the IRON-CRT study, where the most significant difference in left ventricular ejection fraction improvement after iron carboxymaltose treatment was in patients with T-sat <20% [25]. Furthermore, according to a recent study that compared 4 different definitions of ID; using soluble transferrin receptor (sTfR), T-sat and the conventional ferritin-based definition, sTfR and T-sat are the most accurate markers determining prognosis [26].

In our study, observing the conditions of inflammation, CRP did not differ between the non-LVAD and LVAD groups. Ferritin does not significantly correlate with either CRP or serum iron, only ferritin and CRP have a very weak correlation (r > 0.2), which was not statistically significant (*p* = 0.120). However, there was a quite strong and significant correlation between CRP and ferritin in the LVAD subgroup (r > 0.6; *p* = 0.05). Of course, the significance is limited by the low number of patients, but these findings follow the results of a study comparing inflammatory markers in LVAD recipients with healthy subjects and heart failure patients awaiting LVAD implantation found that elevated levels of inflammatory markers persisted even after restoration of cardiac output and organ perfusion [27].

According to recent studies, the presence of LVAD is a condition with endothelial dysfunction and changes in the microvasculature, which subsequently triggers an inflammatory response. The mechanisms involved in the upregulation of inflammatory pathways in patients with LVAD are not yet known, but pathophysiological hypotheses are based on the contact of blood with artificial surfaces and the release of cytokines, especially TNF-alpha, which makes blood vessels fragmented and causes a higher risk of bleeding. Other theories are based on platelet activation at shear stress and the formation of platelet-neutrophil conjugates. The last theory is based on the change of pulsatile flow to continuous flow and RAAS hyperactivity [28].

Last but not least, it is also interesting to compare our results with a recent trial by Veenis et al. that documented the frequency of ID in patients over time—before OTS and LVAD implantation and then at intervals after these procedures. In that study, the incidence in patients awaiting heart transplantation was slightly lower than in ours—in that study, ID was present in 71%. Interestingly, it rose to 77% after heart transplantation. In contrast, the incidence of ID in patients after LVAD implantation was higher than was seen in our small group. The percentage reported by Veenis was 71% and was the same as in patients without LVAD implanted [29]. Of course, we are aware of possible bias given the small size of our patient group. Moreover, due to the fact that transferrin saturation is also influenced by the current state of the organism or the diurnal rhythm, other markers should be investigated. Some recent studies show that the soluble transferrin receptor (sTfR) appears to be one of the most accurate markers [1].

Furthermore, our study verified, that ID may not be expressed by anaemia. In group without LVAD implanted, only 24% of whole population had anaemia and the median of haemoglobin level was the same (133 g/L) in both groups–regardless of the presence of ID by definition. This is an important reminder, that in diagnostics of ID it is not enough to follow the blood count only. Anaemia in patients with LVAD implanted was present in 100% despite the fact, that ID was present only in 57% of this patient’s group.

ID in patients with advanced heart failure is not related to renal insufficiency. In our study, the results showed that there is not a statistically significant difference between creatinine of patients with ID (median 108 μmol/L) and without ID, where the levels were paradoxically slightly higher (median 120.5 μmol/L, *p*-value 0.570).

In the LVAD group, the creatinine levels were lower than in non-LVAD group in general, which is probably caused by better perfusion of kidneys. In this subgroup, there was also no significant difference in creatinine value between groups by iron status (median 100.5 μmol/L for ID, 80 μmol/L for no ID, *p*-value 0.269), but again the subanalysis in the LVAD group has several limitations because of the low power of the test.

In our study, a correlation between ID and the level of NT-proBNP was not confirmed (correlation coefficient was −0.03 for ferritin and −0.2 for T-sat) as known from previous studies [30,31].

There was no significant difference in left ventricular ejection fraction between patients with or without ID. According to the current literature, studies to evaluate the impact of ID on left ventricular function by myocardial strain assessment by echocardiordiography or, better by magnetic resonance imaging, are underway. Similarly, there was no difference in NYHA class; more accurate assessment of performance would be appropriate, for example spiroergometry, 6 MWT or standardized self-assessment of quality of life.

Our results support the importance of monitoring the prevalence of ID in patients with advanced heart failure as mentioned in the 2021 Guidelines for the diagnosis and treatment of acute and chronic heart failure. According to these recommendations, iron supplementation with i.v. ferric carboxymaltose should be considered in patients with LVEF <45% to improve symptoms, performance and quality of life and to reduce rehospitalizations for heart failure in patients with LVEF <50% recently hospitalized for worsening heart failure. Results of further studies exploring the effect of iron carboxymaltose on mortality and the effect in the group of patients with heart failure with preserved ejection fraction are awaited.

## 6. Study Limitations

This study has several limitations. Study was designed like an observational retrospective study. Laboratory parameters were taken in various clinical situations. This may cause, for example, bias in the evaluation of the NT-pro-BNP relationship with the presence of ID. Next, it is only small short-term single centre study with limited number of patients included, especially in LVAD group, which severely limits the statistical power of subgroup analyses.

## 7. Conclusions

Iron deficiency was present in majority of patients with advanced heart failure and was not always accompanied by anaemia. ID in these patients is not related to renal insufficiency.

Our results raise the hypothesis that ferritin-based assessment of iron deficiency may be inaccurate in patients with LVAD due to higher ferritin levels caused by the presence of chronic low-grade inflammation.

Therefore, T-sat seems to be a more accurate parameter to diagnose ID. It also points the need to find new parameters for ID detection like sTfR, to improve the detection and refine the indications of iron supplementation in patients with heart failure.

## Figures and Tables

**Table 1:** Characteristics of patients in the study. WL—waiting list, OHT—orthotopic heart transplantation, LVAD—left ventricle assist device, LVEF—left ventricular ejection fraction; NT-proBNP—N-terminal pro-B-type natriuretic peptide; NYHA—New York Heart Association, HF—heart failure. Values of the parameters with normal distribution are presented as mean ± standard deviation, those with different distribution are in italics and shown as median (lower–upper quartile), and binary variables are expressed as number (percentage of total).

Patients (N)	WL for OHT-Non-LVAD (45)	LVAD Group ^a^ (7)	*p*-Value
**Iron deficiency, *n* (%)**	**37 (82)**	**4 (57)**	**0.154**
Age at inclusion, years *median* (IQR)	56.1 ± 10.8	54.3 ± 5.6	0.673
Gender, women, *n* (%)	12 (27)	0 (0)	<0.001
**CV characteristics**			
NYHA Class IV vs. III, *n* (%)	8 (18)	2 (28.5)	0.574
LVEF, %	20 ± 10	16 ± 5	0.338
**Laboratory parameters**			
NT-proBNP, ng/mL	*4781 (2747–7672)*	*1728 (1138–2361)*	*0.007*
Creatinine, μmol/L	*112 (92–144)*	*90 (68–105)*	*0.049*
Chronic renal insufficiency	29 (64%)	2 (29%)	0.10
Haemoglobin, g/dL	136 ± 17	106 ± 9	<0.001
Anemia	11 (24%)	7 (100%)	<0.001
Ferritin, μg/L	*95 (62–152)*	*268 (106–368)*	*0.019*
Transferrin saturation	0.18 ± 0.09	0.14 ± 0.04	0.272
Serum iron	*10.9 (8.3–14.6)*	*7.8 (5.6–9.8)*	*0.006*
C-reactive protein	*6.0 (3.1–11.0)*	*7.8 (3.9–9.8)*	*0.58*

^a^ patients on waiting list for OHT with LVAD implanted.

**Table 2:** Characteristics of patients without LVAD implanted according to iron status. WL—waiting list, OHT—orthotopic heart transplantation, LVAD—left ventricle assist device, LVEF—left ventricular ejection fraction; NT-proBNP—N-terminal pro-B-type natriuretic peptide; NYHA—New York Heart Association, HF—heart failure, CM—cardiomyopathy, Values are median (average ± standard deviation) unless otherwise specified.

Patient Groups by Iron Status *n* (%)	WL for OHT-Non-LVAD ^a^ (45)			*p*-Value
	All	ID *37* (82%)	No ID *8* (18%)	
Age at inclusion, years	56.1 ± 10.8	55.8 ± 11.3	56.5 (56.1 ± 7.6)	0.981
Gender, women *n* (%)	12 (27%)	12 (32%)	0 (0%)	0.048
**CV characteristics**				
NYHA Class IV vs. III, *n* (%)	8 (18%)	6 (16%)	2 (25%)	0.590
LVEF, %	20 ± 10	21 ± 9	19 ± 13	0.646
**Laboratory parameters**				
NT-proBNP, ng/mL	*4781 (2747–7672)*	4781 (2747–6989)	5963 (2518–7913)	0.767
Creatinine, μmol/L	112 (92–144)	121 (109–144)	108 (91–144)	0.570
Chronic renal insufficiency	29 (64%)	22 (59%)	7 (88%)	0.23
Haemoglobin, g/dL	136 ± 17)	136 ± 16	136 ± 23	0.976
Anemia	11 (24%)	*8 (22%)*	*3 (38%)*	*0.38*
Ferritin, μg/L	95 (62–152)	87 (58–108)	269 (157–362)	<0.001
Transferrin saturation	0.18 ± 0.09	0.15 ± 0.06	0.30 ± 0.10	<0.001
Serum iron	10.9 (8.3–14.6)	9.9 (7.9–12.7)	15.7 (13.4–27)	0.001
C-reactive protein	6.0 (3.1–11.0)	6.5 (3.1–11.0)	3.8 (3.2–7.0)	0.72

^a^ patients on waiting list for OHT without LVAD implanted.

## Data Availability

The data presented in this study are openly available in FigShare at https://doi.org/10.6084/m9.figshare.21432612, accessed on 2 October 2022.
